# Spatially Annotated Single Cell Sequencing for Unraveling Intratumor Heterogeneity

**DOI:** 10.3389/fbioe.2022.829509

**Published:** 2022-02-22

**Authors:** Myrthe M. Smit, Kate J. Feller, Li You, Jelle Storteboom, Yasin Begce, Cecile Beerens, Miao-Ping Chien

**Affiliations:** ^1^ Department of Molecular Genetics, Erasmus University Medical Center, Rotterdam, Netherlands; ^2^ Erasmus MC Cancer Institute, Rotterdam, Netherlands; ^3^ Oncode Institute, Utrecht, Netherlands

**Keywords:** spatial transcriptomics, single cell sequencing, functional single cell sequencing, intratumoral heterogeneity, epithelial-to-mesenchym transition (EMT)

## Abstract

Intratumor heterogeneity is a major obstacle to effective cancer treatment. Current methods to study intratumor heterogeneity using single-cell RNA sequencing (scRNA-seq) lack information on the spatial organization of cells. While state-of-the art spatial transcriptomics methods capture the spatial distribution, they either lack single cell resolution or have relatively low transcript counts. Here, we introduce spatially annotated single cell sequencing, based on the previously developed functional single cell sequencing (FUNseq) technique, to spatially profile tumor cells with deep scRNA-seq and single cell resolution. Using our approach, we profiled cells located at different distances from the center of a 2D epithelial cell mass. By profiling the cell patch in concentric bands of varying width, we showed that cells at the outermost edge of the patch responded strongest to their local microenvironment, behaved most invasively, and activated the process of epithelial-to-mesenchymal transition (EMT) to migrate to low-confluence areas. We inferred cell-cell communication networks and demonstrated that cells in the outermost ∼10 cell wide band, which we termed the invasive edge, induced similar phenotypic plasticity in neighboring regions. Applying FUNseq to spatially annotate and profile tumor cells enables deep characterization of tumor subpopulations, thereby unraveling the mechanistic basis for intratumor heterogeneity.

## Introduction

Intratumor heterogeneity, both at the genetic and transcriptomic level, is commonly observed in various cancer types and complicates diagnosis and treatment (Gerlinger et al., 2012; Patel et al., 2014; Morrissy et al., 2017; Puram et al., 2017; Berglund et al., 2018). Rare populations of cells can contribute to increased tumor progression (Burrell et al., 2013; Patel et al., 2014), metastatic potential (Yachida et al., 2010; Navin et al., 2011) and therapy resistance (Sottoriva et al., 2013; Patel et al., 2014; Tirosh et al., 2016). Single-cell sequencing is key to characterizing the complexity of intratumor heterogeneity, but lacks information about functional properties and spatial organization of cells (Lawson et al., 2018). We have recently developed a functionally annotated transcriptomic profiling technique, called functional single cell sequencing (FUNseq), to study heterogeneous populations of tumor cells based on functional features (You et al., 2021). This technology uses live-cell imaging to identify cells with a phenotype of interest (e.g., cell migration or morphology), which can then be phototagged (*via* a photopatterned device) with a photoactivatable dye, isolated and subjected to single-cell RNA sequencing (scRNA-seq). Hence, FUNseq links phenotypic traits to gene expression profiles of rare subpopulations of tumor cells, thereby identifying the underlying mechanisms of intratumor heterogeneity. However, cells are currently labeled using a single dye, making it impossible to discern cells based on their spatial organization.

Here, we applied FUNseq to characterize intratumor heterogeneity in tumor subpopulations that are spatially located differently in an untransformed, mammary epithelial tumor model. We specifically focused on the epithelial-to-mesenchymal transition (EMT), as this is an important source for intratumor heterogeneity (Nieto et al., 2016). During EMT, epithelial cells gradually acquire a mesenchymal phenotype, thereby losing their cell-cell adhesion and cell polarity while gaining the ability to migrate and invade (Nieto et al., 2016; Pastushenko et al., 2018; Revenco et al., 2019). EMT can be induced by multiple stimuli, including various growth factors and a cell’s local microenvironment (Cook and Vanderhyden 2020). To illustrate, cells at the migrating front of tumors express higher levels of EMT marker genes than cells in the center (Puram et al., 2017). Recently, McFaline-Figueroa et al. (2019) made a similar observation using an *in vitro* tumor model, showing that untransformed MCF10A cells in the outer layer of a high-confluence patch of cells undergo EMT. However, the exact transcriptomic changes that cause this EMT are currently unknown. To identify the genes that drive the outward migration, one needs to profile the cells in the outermost layer of the cell patch (i.e., the invasive edge). This could be done by spatially annotating bands of cells before subjecting them to scRNA-seq, which enables specific characterization of the invasive edge.

Using a similar tumor model as McFaline-Figueroa et al., we applied FUNseq to profile MCF10A epithelial cells that were spatially located in the outer layer (∼1,000–1,500 μm bandwidth, ∼50 cell wide band) or the outermost layer (250 μm bandwidth, ∼10 cell wide band) of the cell mass. We demonstrated that cells in the outermost layer were progressing through EMT and induced similar phenotypic plasticity in neighboring regions. Using cell-cell communication network analysis, we also showed that the Ephrin, EGF and VEGF signaling pathways were involved in driving this invasive behavior. Our data indicates that FUNseq can spatially profile intratumor heterogeneity, thereby unraveling the underlying mechanisms for the observed phenotypic variations.

## Results

### FUNseq Can Spatially Annotate and Profile Cells With Desired Spatial Bandwidths

**FIGURE 1:**
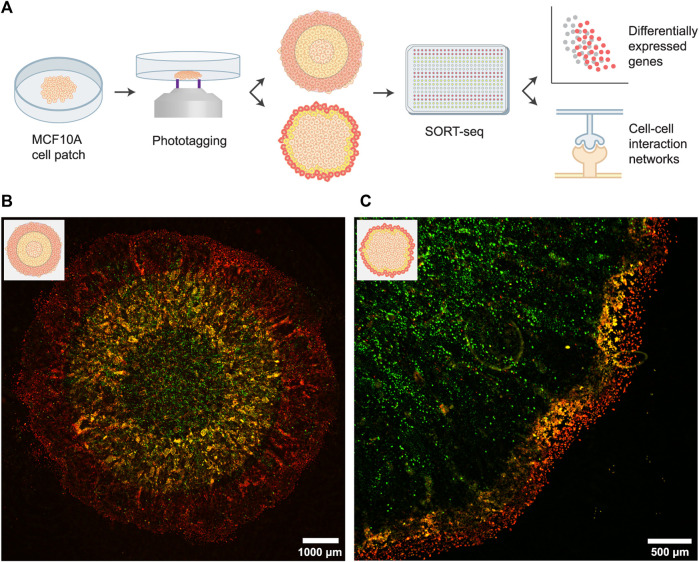
Spatially profiling an *in vitro* tumor model using the FUNseq technology. **(A)** Schematic depiction of the assay, cell labeling and scRNA-seq analysis. For the cell labeling (middle panel), we either phototagged concentric rings of equal width (top; 1,000–1,500 μm bandwidth) or 250 μm wide bands at the invasive edge (bottom). In both approaches, the outer population was labeled with JF646 phototagging dye (red) and the middle population was labeled with both JF549 and JF646 (yellow). **(B)** Patch of MCF10A cells expressing a GFP marker that was phototagged with the larger bandwidth. Green: GFP, yellow: JF549, red: JF646. **(C)** Phototagging the invasive edge of a MCF10A cell patch yields well-demarcated bands of cells.

We applied FUNseq to profile spatial heterogeneity in an *in vitro* tumor model: untransformed, mammary epithelial MCF10A cells (Figure 1A). MCF10A cells expressing a GFP marker were seeded in a high-confluence, circular patch. After growing the cells for 6 days, cells at the leading edge of the patch acquired a spindle-like morphology and migrated to unoccupied areas of the dish (Supplementary Figure S1), indicating that they might have undergone EMT (Vuoriluoto et al., 2011).

Next, we imaged the cells using our custom-built Ultrawide Field-of-view Optical (UFO) microscope (You et al., 2021) and identified the outer, middle and inner regions (with a bandwidth of 1,000–1,500 μm) of the patch. Cells were first incubated with photoactivatable Janelia Fluor 646 (JF646) dye, after which we phototagged the outer one-third of the patch (Figure 1B; cells emit red fluorescence (λ_ex_: ∼650 nm, λ_em_: ∼665 nm) after photoactivation). Subsequently, we incubated cells with photoactivatable Janelia Fluor 549 dye (JF549) and phototagged the middle one-third of the patch (cells emit green fluorescence (λ_ex_: ∼550 nm, λ_em_: ∼570 nm) after photoactivation). Hence, cells in the middle ring were labeled with both dyes, as the cytoplasmic JF646 dye is retained within cells. Labeled populations were isolated by flow cytometry and sequenced using SORTseq, a plate-based, modified CEL-seq2 scRNA-seq technology (Hashimshony et al., 2016; Muraro et al., 2016).

A similar labeling strategy can be used to profile the invasive edge at a higher resolution. For this, we phototagged cells in the outermost layer (250 μm bandwidth, ∼10 cell wide band) of the patch with JF646 and we phototagged cells in the next 250 μm with both JF549 and JF646. Live-cell imaging of the labeled patches showed well-demarcated bands of cells (Figure 1C), validating that FUNseq can be used to annotate and isolate confined tumor regions with desired spatial bandwidth.

### FUNseq Identified Subtle Variations in Gene Expression Profiles Between Tumor Regions

**FIGURE 2:**
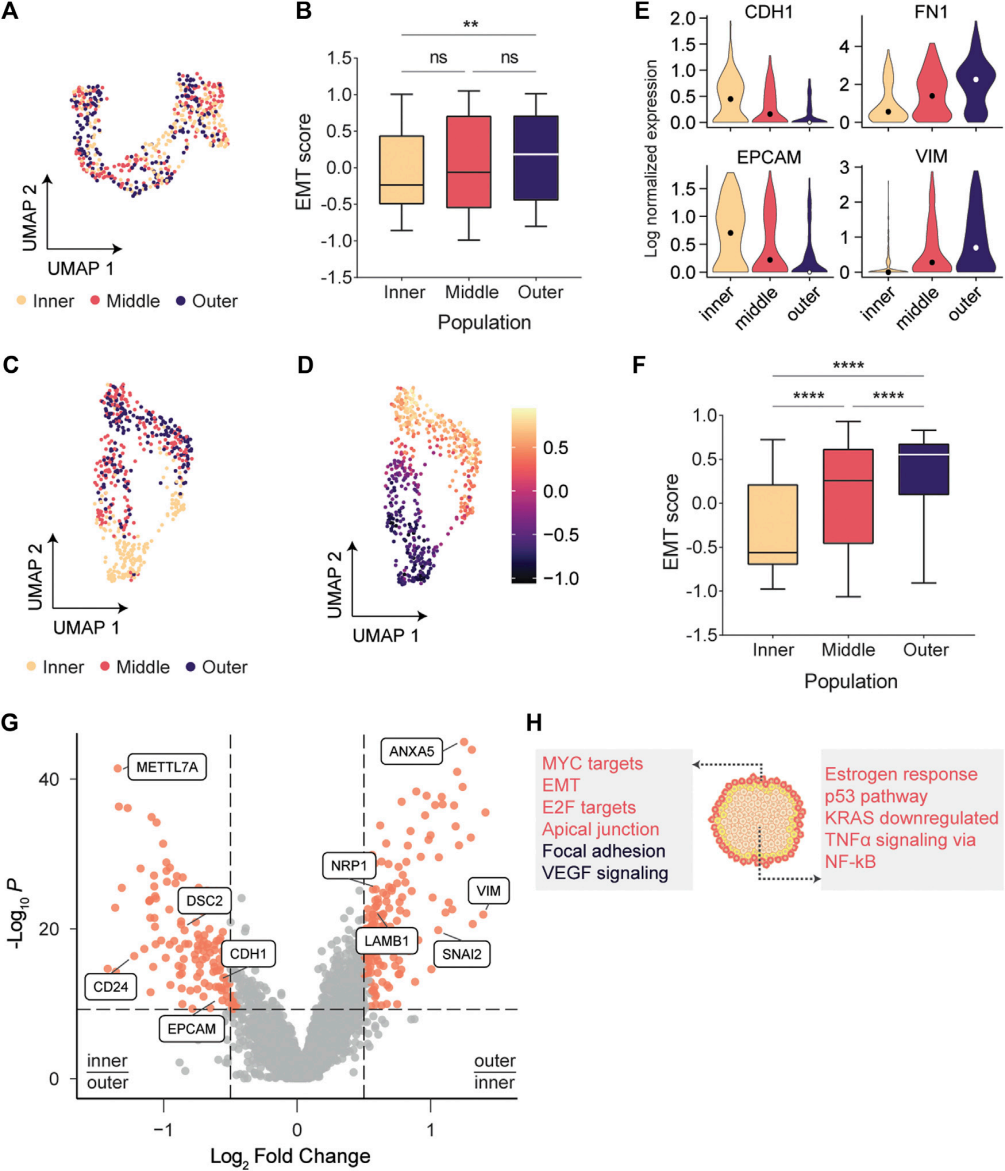
scRNA-seq indicated that cells at the invasive edge were progressing through EMT. **(A)** UMAP embedding of cells labeled with the larger bandwidth showed a modest separation of tumor regions, but no coherent clusters were formed. **(B)** EMT scores between inner and outer populations vary significantly (*p* = .0017; Kruskal-Wallis test). **(C)** Inner and outermost tumor regions labeled with the smaller bandwidth separate clearly in UMAP space. **(D)** EMT scores gradually increase across the UMAP embedding. **(E)** Expression of classic epithelial markers decreases radially outwards while expression of classic mesenchymal markers increases. **(F)** EMT scores are significantly varying between adjacent populations (*p* < .0001; Kruskal-Wallis test). **(G)** Volcano plot indicating genes overexpressed in the outermost population (log_2_(FC) > .5) and in the inner population (log_2_(FC) < −.5). **(H)** Overrepresentation analysis using the MSigDB Hallmarks (red) and Wikipathways (black) databases.

To couple the observed phenotypic plasticity in the outer layer to underlying transcriptomic changes, tumor subpopulations were subjected to scRNA-seq. We sequenced two biological replicates of patches phototagged with the larger bandwidth, yielding a total of 743 analyzed single cell transcriptomes (Supplementary Figure S2). Dimensionality reduction using Uniform Manifold Approximation and Projection (UMAP) (McInnes et al., 2018) indicated a modest separation of the populations but did not form coherent clusters (Figure 2A), suggesting that there is substantial similarity of the gene expression profiles between the tumor regions.

To quantity the level of EMT in each subpopulation, we calculated EMT scores using Gene Set Variation Analysis (GSVA) (Hänzelmann et al., 2013). For each cell, an epithelial (E) and mesenchymal (M) score was calculated using two gene sets containing 65 epithelial and 115 mesenchymal genes (Cesano 2015). Following the approach of Sacchetti et al. (2021), we subtracted the E score from the M score to define a single EMT score for each cell (EMT = M – E). Cells in the outer layer had significantly higher EMT scores than cells in the center (Kruskal-Wallis test, *p* = .0017; Figure 2B). However, no significant changes between adjacent populations were observed, presumably because the relatively large number of cells per region led to substantial heterogeneity within each population (Supplementary Figure S3). This solidified our notion that one needs to specifically profile the invasive edge to reliably identify the transcriptomic drivers for migration and invasion. Hence, we next sought to profile the migrating front at a higher resolution.

### Cells at the Invasive Edge Strongly Activated the Epithelial-to-Mesenchymal Transition

We phototagged the migrating front (∼10 cell wide bands) and separated the outermost cells from the inner tumor mass (Figure 1C). Using this high-resolution phototagging approach, we analyzed 696 single cell transcriptomes from two biological replicates. Dimensionality reduction now revealed coherent clusters of cells that segregate based on the spatial populations (Figure 2C). The middle and outermost layer clustered together in the UMAP embedding, presumably since cells in both layers are progressing through EMT. Classically, EMT has been viewed as a discrete process in which cells pass through distinct transition stages before acquiring a fully mesenchymal morphology (Pastushenko and Blanpain 2019). Our UMAP embedding (Figure 2D) indicated that EMT scores vary continuously across the embedded cells, further solidifying recent findings that EMT is a continuous process (McFaline-Figueroa et al., 2019; Cook and Vanderhyden 2020). Expression of the classic epithelial markers E-cadherin (CDH1) and EPCAM gradually decreased from the center to the edge of the patch, while the mesenchymal markers VIM and FN1 showed a reciprocal pattern, suggesting that cells are exhibiting epithelial-mesenchymal plasticity (Zhao et al., 2015; Yang et al., 2020) (Figure 2E; Supplementary Figure S4). These changes in CDH1 expression were not detected by McFaline-Figueroa et al. (2019), underscoring the value of deep sequencing using FUNseq to resolve subtle transcriptomic changes. Moreover, we found that adjacent populations have significantly varying EMT scores (*p* < .0001; Figure 2F), further increasing our confidence that profiling the invasive edge of the tumor model could identify drivers of migration and invasion.

Next, we identified differentially expressed genes (DEGs) between the subpopulations and found that classic mesenchymal markers such as VIM and the EMT transcription factor SNAI2 were upregulated in the outermost population, while the epithelial markers CDH1 and EPCAM were upregulated in the center of the patch (Figure 2G; Supplementary Figure S4). Genes upregulated in the outermost (*n* = 165 DEGs) or center (*n* = 142 DEGs) population were used for overrepresentation analysis using the MSigDB Hallmarks gene set collection (Liberzon et al., 2015) and the Wikipathways database (Martens et al., 2021) (Figure 2H). As expected, genes involved in EMT and extracellular matrix interactions were overrepresented in the outermost population. Additionally, cells at the invasive edge were enriched for the vascular endothelial growth factor (VEGF) signaling pathway, which can induce cell migration and EMT (Anthony D. Yang et al., 2006; Gonzalez-Moreno et al., 2010; Bhattacharya et al., 2017). VEGF can activate the neuropilin-1 receptor (NRP1), which is upregulated in the outermost population (Figure 2G; Supplementary Figure S5) and promotes proliferation, migration and invasion of tumor cells (Goel and Mercurio 2013; Luo et al., 2016).

### Cell-Cell Communication Network Analysis Identified Multiple Epithelial-to-Mesenchymal Transition Inducers

**FIGURE 3:**
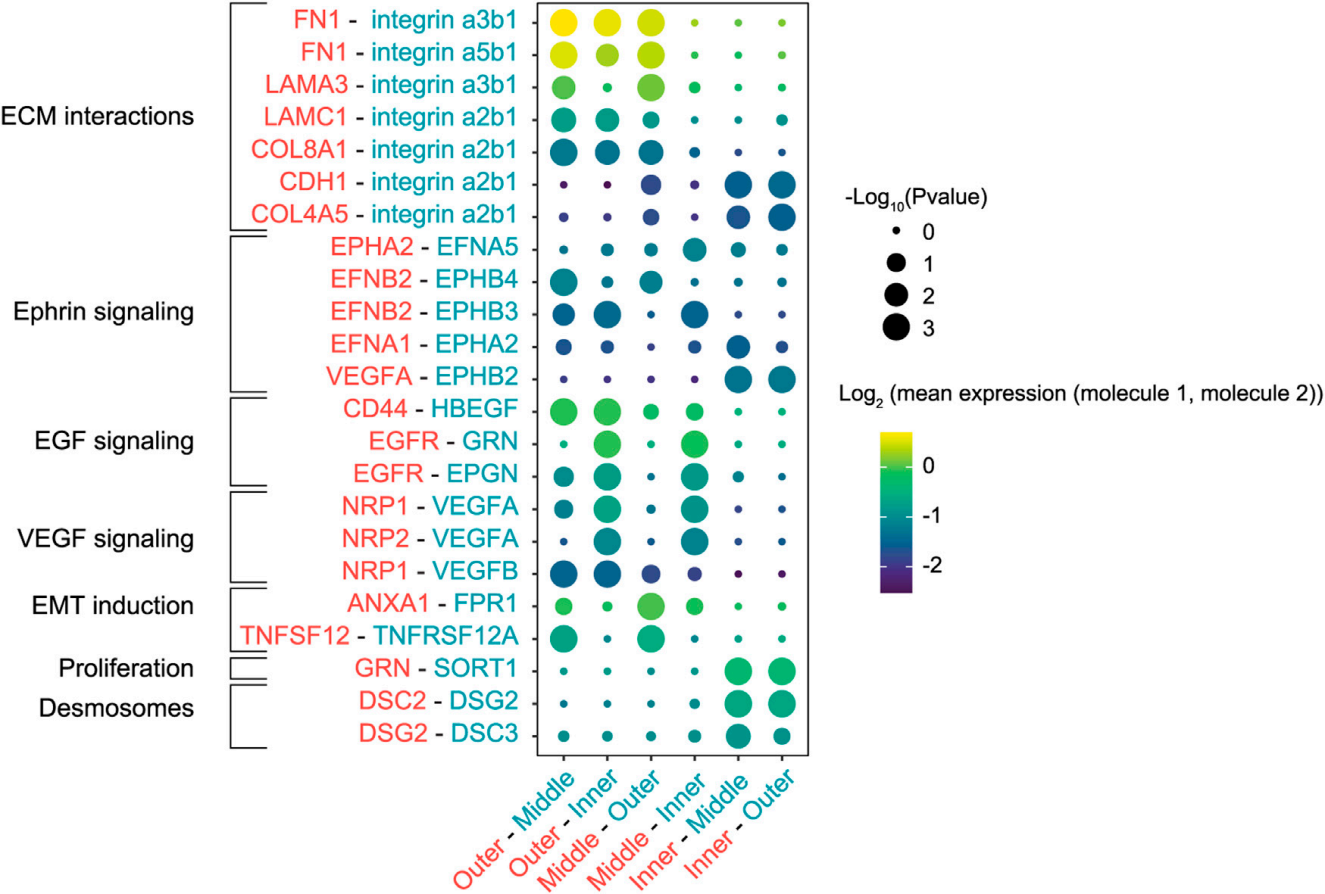
Cell-cell interactions between cells in various patch regions labeled with the smaller bandwidth. Interactions were inferred based on the expression of ligands and receptors in the different cell populations. The first molecule in each interaction pair (rows) corresponds to the first region in each population pair (columns). Circles scaled by the significance of the interaction and colored by the average expression level of ligand and receptor.

Since a wide range of transcription factors and extracellular stimuli are involved in stimulating EMT (Nieto et al., 2016), we next set out to map the cell-cell communication networks that regulate the EMT in our *in vitro* tumor model. We re-analyzed scRNA-seq data from the high-resolution labeling experiment to identify interactions between the different populations using CellPhoneDB, a repository of ligand-receptor complexes that can predict enriched cellular interactions based on the expression of ligands and receptors in cell populations (Efremova et al., 2020). The outermost population was highly enriched for fibronectin (FN1), laminin (LAMA3 and LAMC1) and collagen (COL8A1 and COL4A1) expression, extracellular matrix (ECM) proteins that can interact with the integrins expressed in the middle and inner populations (Figure 3). Specifically, interactions of fibronectin and laminin with the α3β1 integrin modulate cell adhesion to the ECM and cell motility (Meng et al., 2009; Hamill et al., 2010; Jia et al., 2010; Zhang et al., 2017). Interestingly, this analysis predicted multiple interactions in the Ephrin-signaling pathway, in which ligands and receptors activate bidirectional signals that can lead to somewhat paradoxical downstream effects (Pasquale 2008). To illustrate, cells in the outermost and middle populations expressed the EphB4 receptor and its ligand EphrinB2 (EFNB2) (Supplementary Figure S6). Activation of EphB4 induces cell migration and invasion in cancer cells (Steinle et al., 2002; Kumar et al., 2006; Nai-Ying Yang et al., 2006), although the exact opposite effect has also been reported (Noren et al., 2006). Additionally, reverse signaling through EphrinB2 can stimulate cell migration through the PI3K pathway (Steinle et al., 2002; Kumar et al., 2006).

Finally, CellPhoneDB inferred enrichment of multiple EMT inducers and their receptors in the outermost population, such as tumor necrosis factor (TNFA) and genes involved in the EGF pathway (CD44, EGFR, EPGN, HBEGF) (Cheng et al., 2012; Revenco et al., 2019; Cook and Vanderhyden 2020). Conversely, cells in the center of the patch were enriched for DSC2 and DSG2, genes that encode components of desmosome cell-cell junctions (Garrod and Chidgey 2008; Nekrasova and Green 2013), hallmarks of an epithelial phenotype. Taken together, the identified cell-cell interactions indicated that cells at the migrating front responded to their local microenvironment and stimulated similar invasive behavior in neighboring regions.

## Discussion

Intratumor heterogeneity is a major challenge for effective cancer treatment. Single-cell genomics and transcriptomics proved themselves valuable methods to study this heterogeneity, but lack information about the spatial organization of cells. Recently, various spatial transcriptomics methods have been developed to add positional information from tissue sections to the single-cell transcriptomes (Ståhl et al., 2016; Vickovic et al., 2019; Stickels et al., 2021), providing numerous insights in cancer biology and other fields (Longo et al., 2021). However, these methods either lack single cell resolution or have substantially lower transcript counts per cell than conventional scRNA-seq. Here, we applied our recently developed FUNseq technology to spatially profile confined tumor regions. The strength of this method lies in the combination of labeling tumor regions guided by live-cell imaging and deep sequencing of single cells. This allowed us to profile gene expression in isolated tumor regions using 34,000 transcripts per cell (Supplementary Figure S2), compared to the 494 and 11.5 transcripts per 10 μm bead for Slide-seq V2 and HDST, respectively (Stickels et al., 2021). The increased sensitivity of FUNseq allows us to study low abundance transcripts, enabling deep characterization of tumor cells.

We profiled tumor heterogeneity in an *in vitro* tumor model (McFaline-Figueroa et al., 2019) by annotating cells located at different distances from the center of a 2D epithelial cell mass. Cells in the outermost layer or invasive edge (∼10 cell wide band) of this patch were progressing through EMT, suggesting that these cells sense their local microenvironment and acquire a mesenchymal phenotype to migrate to unoccupied areas of the dish. Taking advantage of the FUNseq’s deep sequencing, we characterized cell-cell interaction networks between the different tumor regions. We identified various interactions between outermost cells and ECM components that can stimulate cell migration and we showed that outermost cells are enriched for ligands and receptors that can stimulate EMT, such as components of the Ephrin, EGF and VEGF signaling pathways.

By combining phototagging of confined tumor regions and deep sequencing of single cells, we characterized the transcriptomic heterogeneity in a population of untransformed epithelial cells. To fully explore the potential of FUNseq, the next step would be to profile tumor sections, which have much higher complexity than relatively homogeneous cell lines. We envision that FUNseq might address important questions about intratumor heterogeneity, such as how tumor cells interact with the tumor microenvironment and how tumor composition affects treatment outcome.

In summary, we demonstrated that FUNseq can spatially annotate and profile subpopulations of an *in vitro* tumor model. We showed that cells at the invasive edge (∼10 cells wide band) of a high-confluence patch of cells underwent EMT, migrated to low-confluence areas and induced similar phenotypic plasticity in neighboring cells. Spatially profiling tumor cells using FUNseq enables deep characterization of intratumor heterogeneity, thereby laying the foundation for a more complete understanding of tumor biology.

## Materials and Methods

### Cell Culture

MCF10A_H2B_GFP human breast epithelial cells were a kind gift of Reuven Agami (Netherlands Cancer Institute). Cells were cultured at 37°C and 5% CO_2_ in DMEM/F12 medium without phenol red (Gibco), supplemented with 5% Donor Equine Serum, 1% penicillin/streptomycin, 20 ng/ml EGF, 500 ng/ml hydrocortisone, 100 ng/ml cholera toxin and 10 μg/ml insulin.

Before conducting experiments, cells were seeded on 20 mm glass bottom dishes (Cellvis), coated with 0.1 mg/ml fibronectin (EMD Millipore). 10,000 cells were seeded in a droplet in the center of the dish, such that a circular patch of cells was formed in the center of the dish. After 4.5 h, dishes were washed with Dulbecco’s PBS (Sigma) to remove non-adherent cells. The patch of cells was then cultured in MCF10A medium at 37°C and 5% CO_2_ for 6 days.

### Imaging and Cell Labeling

Cell labeling was performed on the Ultrawide Field-of-view Optical (UFO) microscope developed previously (You et al., 2021). Cells were incubated with 40 µM photoactivatable Janelia Fluor 646 (JF646) dye (Tocris) for 20 min and washed with MCF10A culture medium. Bright-field images were used to localize the patch of cells, after which we identified the cells to be labeled using a low-resolution or high-resolution approach. In the low-resolution tagging approach, we fit three concentric rings with equal bandwidth (1,000–1,500 µm bandwidth) in the area of the patch. In the high-resolution approach, we divide the patch of cells in three layers: the outermost 250 µm of cells (∼10 cell wide band), the next 250 μm, and the inside of the patch.

In both approaches, the outer population of cells was then selectively illuminated for 2 min with 405 nm light using a digital micromirror device (DMD), thereby phototagging these cells with JF646. Next, cells were incubated with 40 µM photoactivatable Janelia Fluor 549 (JF549) dye (Grimm et al., 2016) (Tocris) for 20 min and washed with MCF10A culture medium. The imaging and labeling process was repeated, but now illuminating the middle population of cells. These cells are thus phototagged with JF549 and JF646, as both dyes are present in the cytoplasm and become activated upon illumination. For visualization purposes, image background was subtracted and image contrast was adjusted using ImageJ.

### Cell Isolation and Single-Cell RNA Sequencing

Cells were harvested using trypsin-EDTA without phenol red (Sigma), centrifuged and resuspended in HBSS buffer (Gibco). Live single cells (validated by Draq7 viability staining) were sorted into 384-well plates using the BD FACSMelody Cell Sorter (BD Biosciences), spun-down and stored at −80°C. Library preparation and single-cell RNA sequencing was performed by Single Cell Discoveries (Utrecht, Netherlands) using their custom SORT-seq protocol (Muraro et al., 2016). cDNA libraries were sequenced at 150 k reads/cell on the Illumina NextSeq 500 platform.

### scRNA-Seq Analysis

scRNA-seq data was aligned and preprocessed by Single Cell Discoveries as described by Muraro et al. (2016). Gene expression matrices were processed using Seurat v4 (Hao et al., 2021). Cells containing 2,000–9,000 features and less than 40% mitochondrial genes were selected. Gene expression was either normalized using the SCTransform (Hafemeister and Satija 2019) function for dimensionality reduction, or log-normalized for all other downstream analysis. Cell cycle scoring and regression was performed using a set of G2/M and S phase markers (Tirosh et al., 2016). We performed a Principal Component Analysis (PCA) on the normalized gene expression data and used the first 40 principal components for dimensionality reduction using UMAP.

Differentially expressed genes between the inner and outer populations were identified with Seurat’s findMarkers function using a Wilcoxon rank-sum test and filtering for genes with a Bonferroni corrected *p*-value < 1 × 10^−5^. Genes with log_2_ fold change >0.5 were marked as upregulated and genes with log_2_ fold change <−0.5 were marked as downregulated. Next, overrepresentation analysis (ORA) was performed with the ClusterProfiler v4 package (Wu et al., 2021). The enricher function was used with default settings (one-sided Fisher’s exact test with Benjamini-Hochberg adjusted *p*-values) and the most significantly enriched processes were visualized.

To calculate the level of EMT in each cell, we followed the approach of Sacchetti et al. (2021) Gene Set Variation analysis was performed using the GSVA package (Hänzelmann et al., 2013), where we used a set of EMT markers that is publicly available from the Nanostring nCounter PanCancer Progression Panel (Cesano 2015). This gene set contained 65 epithelial (E) and 115 mesenchymal (M) genes (Supplementary Table S1). For each cell we calculated its GSVA enrichment scores for the epithelial and mesenchymal genes, after which we subtracted the E score from the M score to define the cell’s EMT score.

Enriched ligand-receptor interactions between the different populations of cells were inferred using the CellphoneDB package (Efremova et al., 2020). This analysis uses empirical shuffling to identify enriched ligand-receptor interactions based on the expression levels in the different populations, while requiring that all subunits from heteromeric ligand-receptor complexes are expressed. Log-normalized gene expression matrices were used as input files and the statistical analysis (without subsampling) was performed using a *p*-value threshold of .01 and requiring that at least 20% of the cells in a population expresses a specific ligand-receptor interaction. To identify highly specific interactions between populations, we filtered for interactions with rank ≤.444. In this way, we filtered for ligand-receptor interactions that were significantly enriched in ≤4 population pairs (out of 9 population pairs in our setup). After this initial prioritization of the predicted interactions, we manually selected biologically relevant interactions for visualization.

## Data Availability

TThe raw data used to generate for this study can be found at NCBI’s GEO DataSets site with an ID number of GSE196245.
