# Supplementary material for: Spatially Annotated Single Cell Sequencing for Unraveling Intratumor Heterogeneity
Source: Front Bioeng Biotechnol. 2022 Feb 22;10:829509. doi: 10.3389/fbioe.2022.829509 (PMC8902076; doi:10.3389/fbioe.2022.829509)
Supplement: Supplementary file 1 [file DataSheet1.pdf]

Supplementary Information

Spatially annotated single cell sequencing for unraveling intratumor heterogeneity

Myrthe M. Smit[1,2], Kate J. Feller[1,2], Li You[1,2], Jelle Storteboom[1,2], Yasin Begce[1,2], Cecile Beerens[1,2], Miao-Ping Chien[1,2,3†]

[1]Department of Molecular Genetics, Erasmus University Medical Center, Rotterdam, The Netherlands [2]Erasmus MC Cancer Institute, The Netherlands. [3]Oncode Institute, Utrecht, The Netherlands. †Senior author: m.p.chien@erasmusmc.nl

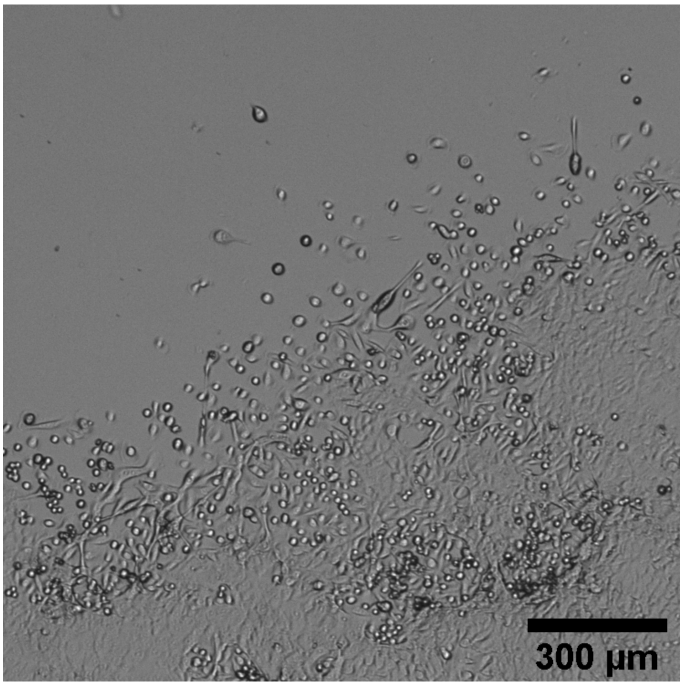

Supplementary Figure 1. *MCF10A cells at the invasive edge of the patch acquire a mesenchymal-like morphology and migrate to low-confluence areas. Brightfield image collected after culturing the high-confluence cell patch for 6 days.*

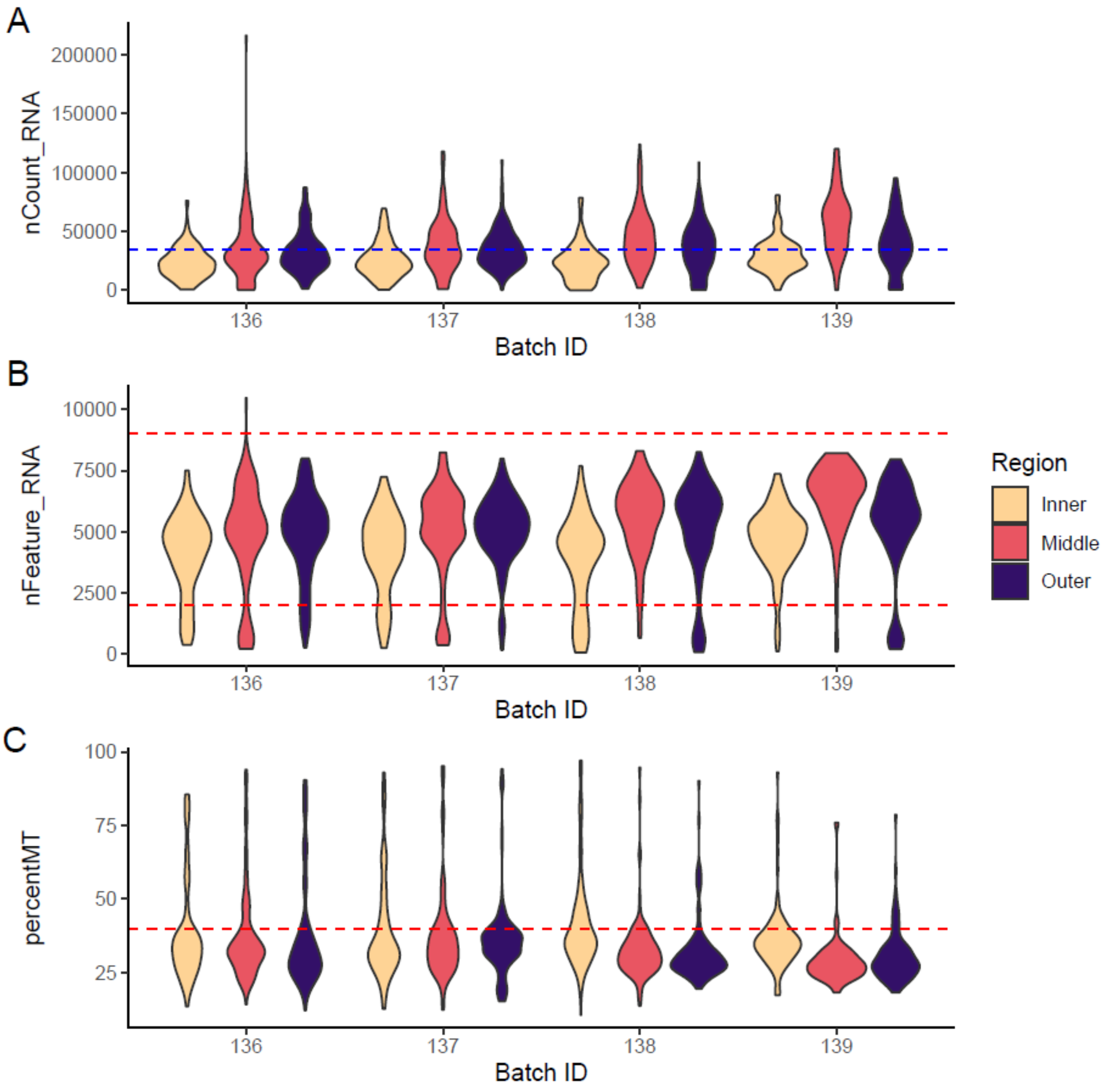

Supplementary Figure 2. Quality control metrics for the scRNA-seq libraries. **A.** Number of Unique Molecular Identifiers (UMIs) per cell. Blue line at 34,492 UMIs per cell indicates the mean number of UMIs in all cells across all libraries. **B.** Number of genes detected per cell. Red lines at 2000 and 9000 features per cell indicate the thresholds used for filtering. **C.** Percentage of mitochondrial genes per cell. Red line at 40% indicates the threshold used for filtering.

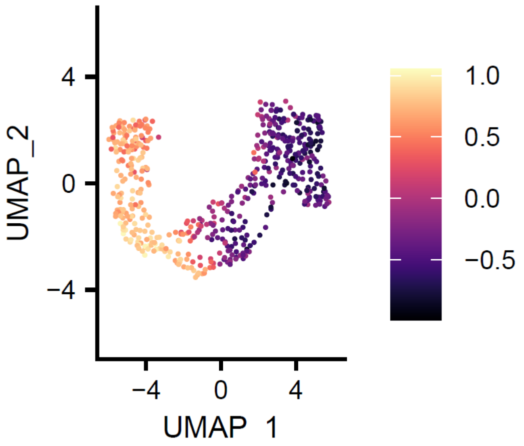

Supplementary Figure 3. EMT scores in the low-resolution tagging approach (~1000-1500 µm bandwidth) form a continuum across the UMAP embedding.

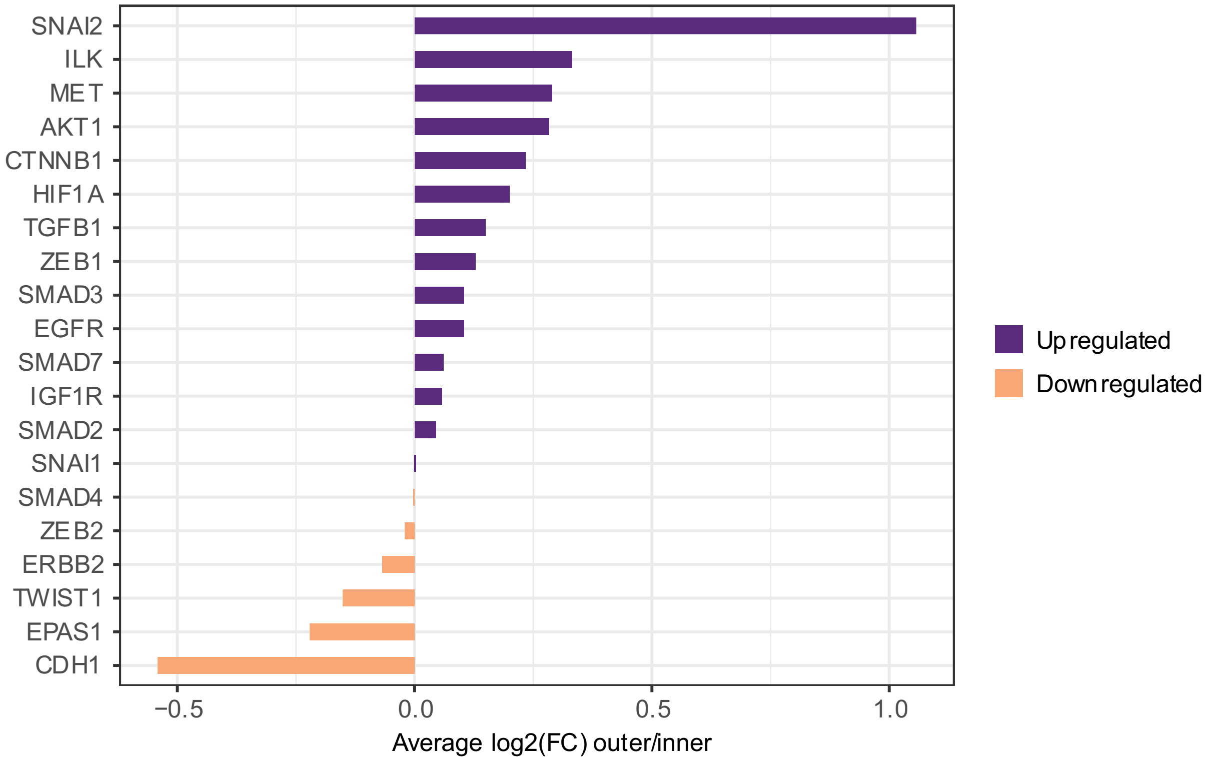

Supplementary Figure 4. Average fold change (log-transformed) of common EMT marker genes and transcription factors (EMT-TFs) when comparing the inner and outermost regions phototagged at the higher resolution (~10 cells wide band). Purple genes are upregulated in the outermost population, orange genes are upregulated in the inner cells. Gene list obtained from Zhao et al. (2015).

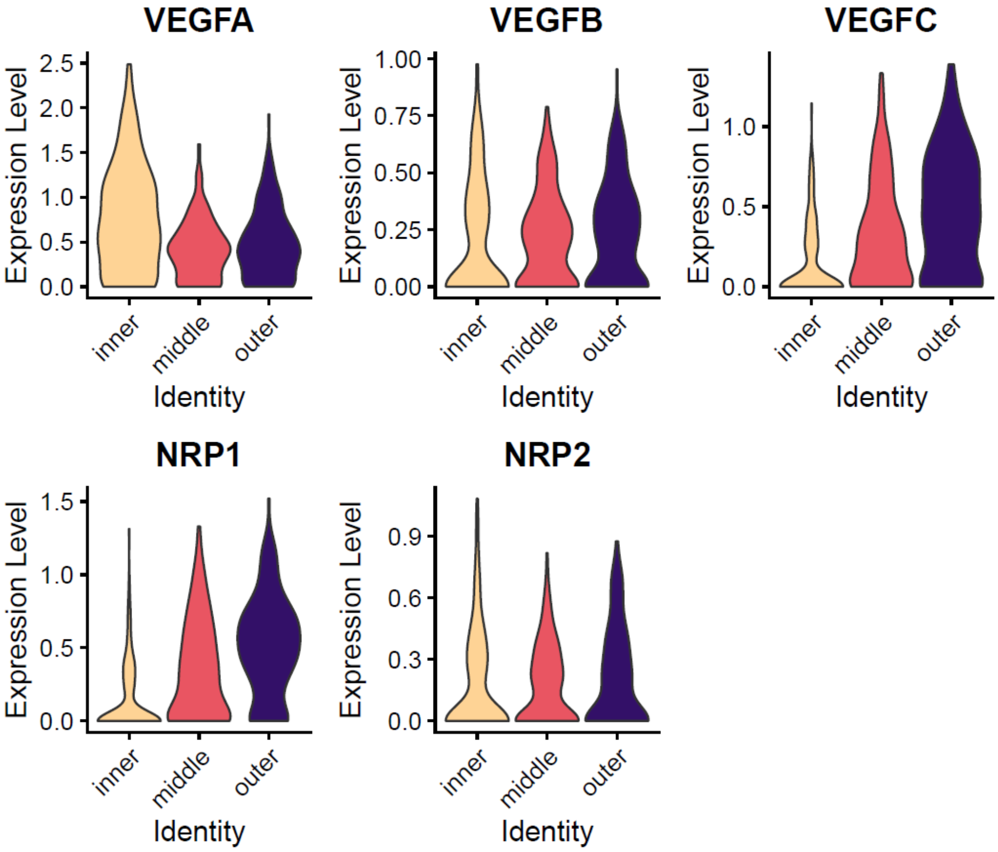

Supplementary Figure 5. Gene expression profile of VEGF ligands and NRP receptors in the high-resolution phototagging approach.

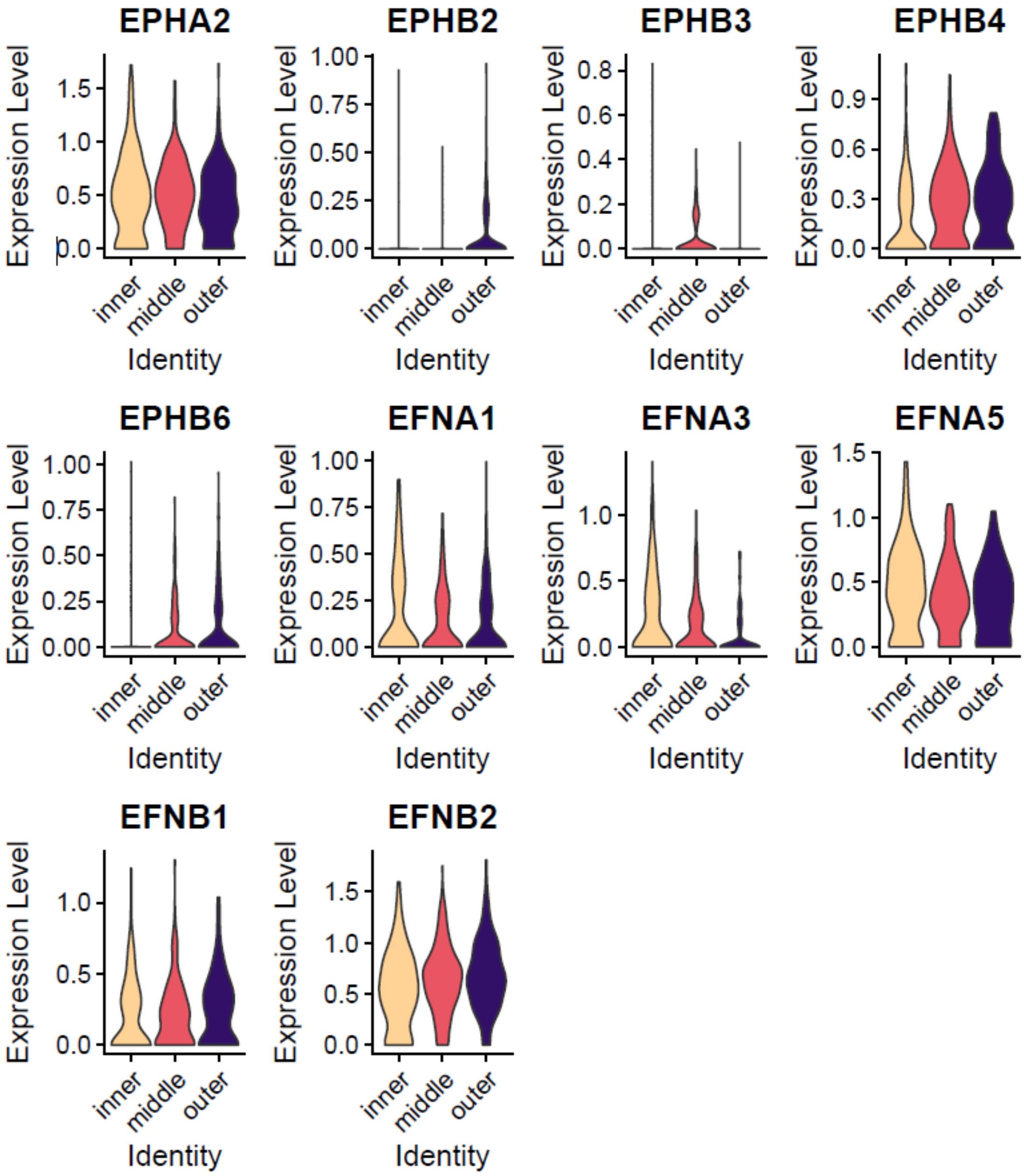

Supplementary Figure 6. Gene expression profiles of Ephrin receptors and ligands in tumor regions phototagged at the higher resolution (~10 cell wide bands).

Epithelial markers			Mesenchymal markers		
AGR2	ESRP1	PTK6	AKAP12	FBLN1	PDGFC
AP1M2	F11R	RAB25	AKAP2	FBN1	PLEKHO1
ARHGAP32	FAM174B	RBM47	AKT3	FERMT2	PLXNC1
BCAS1	FGFR3	S100A14	ANGPTL2	FGL2	PMP22
CBLC	FUT3	SCNN1A	ASPN	FHL1	PTGDS
CD24	GALNT7	SDC4	BGN	FLI1	PTGIS
CD2AP	GDF15	SH3YL1	BICC1	FN1	PTPRC
CDH1	GPR56	SLC44A4	BNC2	FSTL1	PTRF
CDS1	GRHL2	SORD	C1S	FXYD6	PTX3
CEACAM1	HDHD3	SPDEF	CALD1	GIMAP4	QKI
CEACAM5	IRF6	SPINT1	CAV1	GIMAP6	RUNX1T1
CEACAM6	KRT19	ST14	CCL8	GLYR1	SACS
CKMT1A	KRT7	TJP2	CD163	GREM1	SAMSN1
CLDN7	LAD1	TJP3	CDH11	GZMK	SERPINF1
CXADR	MUC1	TMEM30B	CDH2	HEG1	SERPING1
CYB561	MYO5C	TMPRSS2	CDK14	IGF1	SFRP1
ELF3	OCLN	TMPRSS4	CEP170	IL10RA	SLIT2
EPCAM	OVOL2	TOM1L1	CHRDL1	ISLR	SNAI2
EPN3	PLS1	TSPAN1	CLEC2B	ITM2A	SPARC
EPS8L1	PPL	VAMP8	CLIC4	JAM2	SPARCL1
ERBB2	PRR15L	VAV3	COL5A2	JAM3	SRGN
ERBB3	PRSS8		COL6A1	KCNJ8	SYNE1
			COL6A2	KIAA1462	TCF4
			CRISPLD2	LHFP	TNC
			CSF2RB	LOX	TNS1
			CTSK	LY96	TPM2
			CXCL12	MAF	TWIST1
			CXCL13	MEOX2	VCAM1
			CXCR4	MFAP4	VCAN
			CYP1B1	MMP2	VIM
			DCN	MPDZ	VSIG4

			DDR2	MRC1	WIPF1
			DPT	MS4A4A	WWTR1
			DPYSL3	MS4A6A	ZCCHC24
			ECM2	MYLK	ZEB1
			EMP3	NAP1L3	ZEB2
			ENPP2	NR3C1	ZFPM2
			EVI2A	OLFML2B	
			FAP	PCOLCE	

Supplementary Table 1: EMT marker genes from the nCounter PanCancer Progression Panel.
